# Complete chloroplast genome of *Exacum affine* (Gentianaceae): the first plastome of the tribe Exaceae in the family Gentianaceae

**DOI:** 10.1080/23802359.2019.1676672

**Published:** 2019-10-11

**Authors:** Jiuli Wang, Qian Cao, Chuncao He, Yulan Ma, Yinglin Li, Jinxia Liu, Faqi Zhang

**Affiliations:** a Key Laboratory of Biotechnology and Analysis and Test in Qinghai-Tibet Plateau, College of Ecological Environment and Resources, Qinghai Nationalities University, Xining, China;; b Key Laboratory of Adaptation and Evolution of Plateau Biota, Northwest Institute of Plateau Biology, Chinese Academy of Sciences, Xining, China

**Keywords:** *Exacum affine*, plastome, chloroplast, Gentianaceae

## Abstract

*Exacum affine* Balf.f. ex Regel is a traditional medicinal plant in Yemen and also a popular potted plant. In this study, we sequenced the complete chloroplast genome of *E. affine* on the Illumina HiSeq Platform. The plastome sequence is 153,311 bp in length with a typical quadripartite structure, containing a pair of inverted repeated (IR) regions of 26,079 bp that are separated by a large single copy (LSC) region of 83,724 bp, and a small single copy (SSC) region of 17,509 bp. The GC content of the whole cp genome was 43.14%. A total of 132 functional genes were annotated, including 87 protein-coding genes, 37 tRNA genes, and 8 rRNA genes. The complete plastome sequence of *E. affine* will provide genetic and genomic information to promote its horticulture, officinal utilisation and systematics research of Gentianaceae (especially the tribe Exaceae).

*Exacum affine* Balf.f. ex Regel, an annual herb of the tribe Exaceae in the family Gentianaceae, is a traditional medicinal plant in Yemen and also a popular potted plant (Skrzypczak-Pietraszek 2015). The Exaceae represents a small tribe 144–184 species, and the genus *Exacum* consists of about 70 species and distributed in the region of the Indian Ocean and also in southern Asia, the Himalayas, and northern Australia (Skrzypczak-Pietraszek 2015). Chloroplasts are an extremely important organelle to Earth creatures, and their genomes could be smartly engineered to confer useful traits (Jin and Daniell 2015). However, no studies on the complete plastome of the tribe Exaceae have been published. Here, we sequenced the complete chloroplast genome of *E. affine* (Genbank accession number: MK991811) on the Illumina HiSeq Platform.

In this study, the samples of *E. affine* were collected from Daojiao town, Dongguan, Guangdong Province, China (23.03°N, 113.66°E). Total DNA of *E. affine* was extracted from the fresh, young leaves (about 1.5 g) with a modified CTAB method (Doyle and Doyle 1987). The voucher specimen was kept in Herbarium of the Northwest Institute of Plateau Biology, Northwest Institute of Plateau Biology, Chinese Academy of Sciences (HNWP, WJL2018614). Genome sequencing was performed using the Illumina HiSeq Platform (Illumina, San Diego, CA) at Genepioneer Biotechnologies Inc., Nanjing, China. Approximately 7.01 GB of clean data were yielded. The trimmed reads were mainly assembled by SPAdes (Bankevich et al. 2012). The assembled genome was annotated using CpGAVAS (Liu et al. 2012).

The complete chloroplast genome of *E. affine* is 153,311bp in length with a typical quadripartite structure, containing a pair of inverted repeated (IR) regions of 26,079 bp that are separated by a large single copy (LSC) region of 83,724 bp, and a small single copy (SSC) region of 17,509bp. The GC content of the whole cp genome was 43.14%. A total of 132 functional genes were annotated, including 87 protein-coding genes, 37 tRNA genes, and 8 rRNA genes. The protein-coding genes, tRNA genes, and rRNA genes account for 65.90%, 28.03%, and 6.06% of all annotated genes, respectively.

Phylogenetic relationships of *E. affine,* with 20 other species of Gentianaceae and *Rhazyastricta* (Apocynaceae) were resolved by means of Neighbor-joining (NJ). Alignment was conducted using MAFFT (Katoh and Standley 2013). The NJ tree was built using MEGA7 (Kumar et al. 2016) with bootstrap set to 1000. The phylogenetic tree showed that *E. affine* is far from the other species of Gentianaceae that with plastome sequence known (Figure 1). The complete plastome sequence of *E. affine* will provide genetic and genomic information to promote its horticulture, officinal utilisation and systematics research of Gentianaceae (especially the tribe Exaceae).

**Figure 1.**
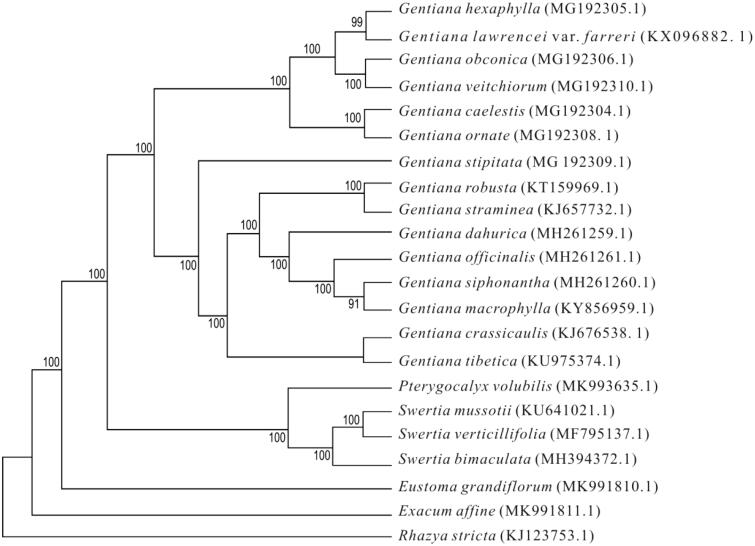
The Neighbor-joining tree based on 22plastome sequences.
